# Development of a Plant Bioactive-Rich Tahini-Based Powder Enriched with Pea Protein and Coconut Milk Powder: Antioxidant Properties, Nutritional Characterization, In Vitro INFOGEST Static Digestion

**DOI:** 10.3390/foods15183268

**Published:** 2026-09-16

**Authors:** Meryem Aparı, Kubra Feyza Erol, Gozde Kutlu

**Affiliations:** 1 Department of Nutrition and Dietetics, Hamidiye Faculty of Health Sciences, University of Health Sciences, 34668 İstanbul, Türkiyekubrafeyza.erol@sbu.edu.tr (K.F.E.); 2 Department of Gastronomy and Culinary Arts, Faculty of Fine Arts, Design and Architecture, Ankara Medipol University, 06050 Ankara, Türkiye

**Keywords:** spray-dried powder, amino acid profile, fatty acid composition, bioaccessibility, phenolic compounds, functional food, antioxidant activity, plant bioactives

## Abstract

This study aimed to develop a spray-dried tahini-based powder enriched with pea protein and coconut milk powder and to comprehensively characterize its plant bioactive composition, antioxidant potential, nutritional properties, and bioaccessibility following the standardized INFOGEST in vitro static gastrointestinal digestion protocol. A stable oil-in-water emulsion containing tahini (50%), pea protein (30%), and coconut milk powder (20%) was prepared by microfluidization and converted into powder by spray drying. The resulting powder exhibited considerable total phenolic content and antioxidant activity, together with distinct amino acid and fatty acid profiles. Simulated gastrointestinal digestion significantly altered the phenolic profile and antioxidant capacity, while several key bioactive compounds showed measurable in vitro bioaccessibility in the intestinal fraction. The formulation also contained high levels of protein (52.69%), lipid (26.42%), and dietary fiber (8.28%) with low moisture (2.28%). Phosphorus, potassium, calcium, and magnesium were the predominant minerals, whereas oleic and linoleic acids were the major fatty acids. Overall, the developed spray-dried tahini-based powder represents a nutritionally valuable plant-derived functional ingredient combining antioxidant potential with the bioaccessibility of essential nutrients.

## 1. Introduction

Sesame (*Sesamum indicum* L.) has gained considerable scientific and industrial attention as a nutrient-dense plant resource with substantial potential for functional food applications. Its seeds provide high-quality proteins, dietary fiber, unsaturated fatty acids, vitamins, minerals, and a diverse array of antioxidant phytochemicals, supporting its use in the development of nutritionally enhanced and plant-based food products [1,2,3,4]. Among sesame-derived foods, tahini, a traditional paste produced from roasted and dehulled sesame seeds, is particularly attractive because it concentrates the nutritional and bioactive constituents of sesame in a versatile food matrix [5,6]. In addition to proteins and unsaturated lipids, tahini contains nutritionally relevant minerals such as calcium, iron, magnesium, and zinc, together with sesame lignans, including sesamin, sesamolin, and sesamol, which have been associated with antioxidant, anti-inflammatory, and cardioprotective activities [3,7,8]. These characteristics position tahini as a promising base material for the development of value-added plant-based functional ingredients.

Despite its nutritional potential, the incorporation of tahini into powdered food systems presents important technological challenges. Its high lipid content and viscous, semi-solid structure can limit feed homogeneity and physical stability when combined with protein- and carbohydrate-rich ingredients. These characteristics may also complicate spray drying, where uncontrolled surface fat and insufficient moisture removal can impair powder flowability, wettability, dispersibility, and oxidative stability [9,10,11]. Spray drying remains one of the most widely used technologies for producing stable food powders because of its scalability, relatively low operating cost, and suitability for continuous processing [9,11,12]. However, thermal exposure during drying may compromise susceptible bioactive compounds and consequently affect their retention and gastrointestinal bioaccessibility. Therefore, pretreatment strategies capable of facilitating the preparation of a homogeneous feed and efficient drying are of particular interest for lipid-rich plant matrices.

Microfluidization offers a potentially effective approach to address these limitations. As a high-pressure homogenization technology, microfluidization generates intense shear, turbulence, cavitation, and rapid pressure changes that can reduce particle size and improve dispersion, emulsion structure, and physical stability [10]. These effects may be particularly relevant to tahini-based systems, in which efficient dispersion of lipid and protein components is essential for obtaining a homogeneous feed prior to drying. Nevertheless, the mechanical stresses generated during microfluidization may also modify the structural and physicochemical properties of proteins and other macromolecules, depending on processing conditions [10]. In the present formulation, microfluidization was therefore employed as a processing step prior to spray drying to obtain the feed used for powder production, rather than as an independently evaluated treatment. Previous research has examined spray-dried sesame oil powders, sesame protein-based encapsulation systems, Pickering emulsions, and sesame-derived nanoemulsions [13,14,15]. However, these approaches have largely focused on isolated sesame fractions or specific encapsulation systems, and the application of sequential microfluidization and spray drying to a whole tahini-based, protein-enriched formulation remains insufficiently explored.

The incorporation of complementary plant-derived ingredients may further enhance both the nutritional and technological functionality of tahini-based systems. Pea protein has emerged as an attractive plant protein because of its favorable nutritional profile, relatively high lysine content, digestibility, and low allergenic potential compared with several other plant protein sources [16,17,18]. In addition to increasing protein density, pea protein possesses interfacial functionality that may contribute to the stabilization of lipid-containing dispersions. Its inclusion in a tahini-based formulation may therefore provide both nutritional enrichment and technological support for the formation of a more homogeneous feed prior to microfluidization and spray drying. Coconut milk powder, meanwhile, can provide additional solids and nutrients while contributing characteristic flavor and aroma to plant-based food formulations [19]. Thus, combining tahini, pea protein, and coconut milk powder provides a formulation strategy based on complementary functionality: tahini serves as the lipid- and sesame-bioactive-rich matrix, pea protein contributes protein enrichment and potential interfacial stabilization, and coconut milk powder provides additional solids and sensory attributes. Importantly, the value of this combination lies not simply in the nutritional contribution of each component but in their potential functional complementarity within a single processable plant-based matrix.

The fate of bioactive compounds during processing and digestion is another critical consideration in the development of functional powders. Phenolic compounds, including phenolic acids, flavonoids, and sesame lignans, contribute substantially to the antioxidant potential of sesame and tahini [1,2,3,4,5,6]. Plant-derived ingredients such as pea and coconut-based materials may additionally contribute phenolic constituents and other phytochemicals to the composite matrix [19]. However, the presence of bioactive compounds in the original raw material does not necessarily indicate that these compounds will remain stable or become bioaccessible following processing and gastrointestinal digestion [20]. Thermal treatment, drying, oxidation, interactions with proteins and lipids, and changes in matrix structure can alter the extractability and stability of phytochemicals and consequently influence their gastrointestinal release. Because spray drying can alter the stability and extractability of bioactive compounds through changes in matrix structure and environmental exposure, the bioactive characteristics of the resulting powder and their behavior during gastrointestinal digestion warrant evaluation [11]. Consequently, assessment of the bioactive profile of the resulting powder together with its gastrointestinal bioaccessibility is essential for determining the functional relevance of newly developed plant-based powders.

Against this background, the present study investigated a novel tahini-based plant protein formulation consisting of tahini (50%), pea protein (30%), and coconut milk powder (20%), processed sequentially by microfluidization and spray drying. The study primarily evaluated the nutritional composition, phenolic profile, and antioxidant capacity of the resulting powder and examined how these characteristics changed following standardized in vitro gastrointestinal digestion using the INFOGEST protocol. Mineral, amino acid, and fatty acid profiles were additionally characterized to provide a broader assessment of the nutritional quality of the formulation. Particular attention was given to the gastrointestinal bioaccessibility of selected phenolic constituents, thereby examining their release and potential bioaccessibility following simulated gastrointestinal digestion. The novelty of the present study lies in the development of a tahini-based plant protein formulation containing pea protein and coconut milk powder and its processing by microfluidization followed by spray drying, with subsequent evaluation of its nutritional composition, bioactive profile, antioxidant capacity, and gastrointestinal bioaccessibility. Rather than assessing the individual contribution of microfluidization or spray drying, the study characterized the resulting spray-dried formulation as an integrated processed product and examined how its compositional and bioactive characteristics changed following standardized in vitro gastrointestinal digestion. We hypothesized that the resulting processed formulation would contain relevant nutritional and bioactive constituents and that selected bioactive compounds would remain measurable and potentially bioaccessible following gastrointestinal digestion. This approach provides insight into the nutritional and gastrointestinal behavior of a novel tahini-based plant powder produced through an integrated processing strategy, while avoiding attribution of observed effects to either microfluidization or spray drying individually.

## 2. Materials and Methods

### 2.1. Experimental Protocol and Material

Pea protein powder (Saf Nutrition; manufacturer: Saffoods Gıda San. A.Ş., Istanbul, Türkiye), 100% organic coconut milk powder (brand: Ayhan Ercan; Istanbul, Türkiye; origin: Sri Lanka), and tahini (brand: Hevsel Bahçesi; Diyarbakır, Türkiye) were commercially procured and used as raw materials in the study. All experimental procedures, including formulation, microfluidization, spray drying, and subsequent physicochemical analyses, were conducted at the Department of Nutrition and Dietetics, Hamidiye Faculty of Health Sciences.

### 2.2. Preparation of Pea Protein–Stabilized Tahini Emulsion and Spray-Drying

Plant–based feed mixtures were prepared using a simplified formulation strategy consisting of pea protein (30%, *w*/*w*), coconut powder (20%, *w*/*w*), and tahini (50%, *w*/*w*). These proportions were determined through preliminary laboratory trials to obtain a homogeneous, physically stable, and spray-dryable feed formulation and were subsequently selected as the final formulation for the microfluidization and spray-drying experiments. These proportions were determined through preliminary laboratory trials of different ingredient ratios, considering mixing homogeneity, feed consistency, physical stability, sensory characteristics, and suitability for microfluidization and spray drying. This ratio was subsequently selected as a workable and spray-dryable formulation for the subsequent microfluidization and spray-drying experiments, rather than as a statistically optimized formulation. All components were accurately weighed and mixed for 5 min using a Thermomix (Vorwerk, Wuppertal, Germany) under controlled conditions until a homogeneous mixture was obtained. The mixture was then processed using a high-pressure microfluidizer (M-110Y, Microfluidics Inc., Westwood, MA, USA) to produce a fine oil-in-water emulsion. The emulsion was passed through the microfluidizer three times at 1200 bar, following the procedure described by Sinem et al. [10]. The microfluidized feed was directly subjected to spray drying without any additional pre-heating of the aqueous phase or the use of stabilizing agents. Spray drying was carried out using a mini laboratory spray dryer (B-290, BÜCHI, Flawil, Switzerland) equipped with a two-fluid nozzle (0.7 mm). The water evaporation capacity of the dryer was 1.0 kg H_2_O/h. Prior to atomization, the feed temperature was maintained at 50 °C. The inlet and outlet air temperatures were set at 160 °C and 75 °C, respectively. The pump rate was adjusted to 40%, corresponding to a feed rate of 6.5 mL/min, while the volumetric air flow rate was maintained at 600 L/h, as reported by Liu et al. [21]. The resulting powder was collected, stored under ambient conditions, and subsequently characterized for its nutritional, physicochemical, and bioactive properties before and after simulated gastrointestinal digestion.

### 2.3. Proximate Composition, pH, and Color Characteristics

Proximate composition of the tahini, pea protein powder, coconut milk powder, and protein enriched tahini powder was determined in accordance with standard methods described by AOAC International [22]. Moisture content was measured by drying 3.0 g of each sample in a hot-air oven at 105 °C until constant weight was achieved. Crude protein content was quantified using the Kjeldahl method on 0.5 g samples and calculated as total nitrogen multiplied by a conversion factor of 6.25. Lipid content was determined by Soxhlet extraction using petroleum ether as the solvent. Ash content was obtained by incinerating 5.0 g of sample in a muffle furnace at 550 °C for 6 h. Crude fiber content was determined involving sequential acid and alkaline digestion of the defatted sample, followed by filtration, drying, incineration, and gravimetric quantification of the remaining residue. Total carbohydrate content was calculated by difference, subtracting the sum of moisture, ash, protein, fat, and crude fiber percentages from 100 [23]. Moreover, the pH of samples was measured using a digital pH meter (HI 2002–02, Hanna Instruments, Olsztyn, Poland) after homogenization with distilled water at a ratio of 1:2 (*w*/*v*) [24]. Color characteristics were evaluated using a colorimeter (CR-300 Chroma Meter, Minolta, Osaka, Japan) calibrated against standard black and white reference tiles prior to analysis. The CIELAB color system was employed to determine the L*, a*, and b* parameters, where L* indicates lightness, a* represents the red–green axis, and b* corresponds to the yellow–blue axis [24].

### 2.4. In Vitro Static Digestion with the INFOGEST Model

**Figure 1:**
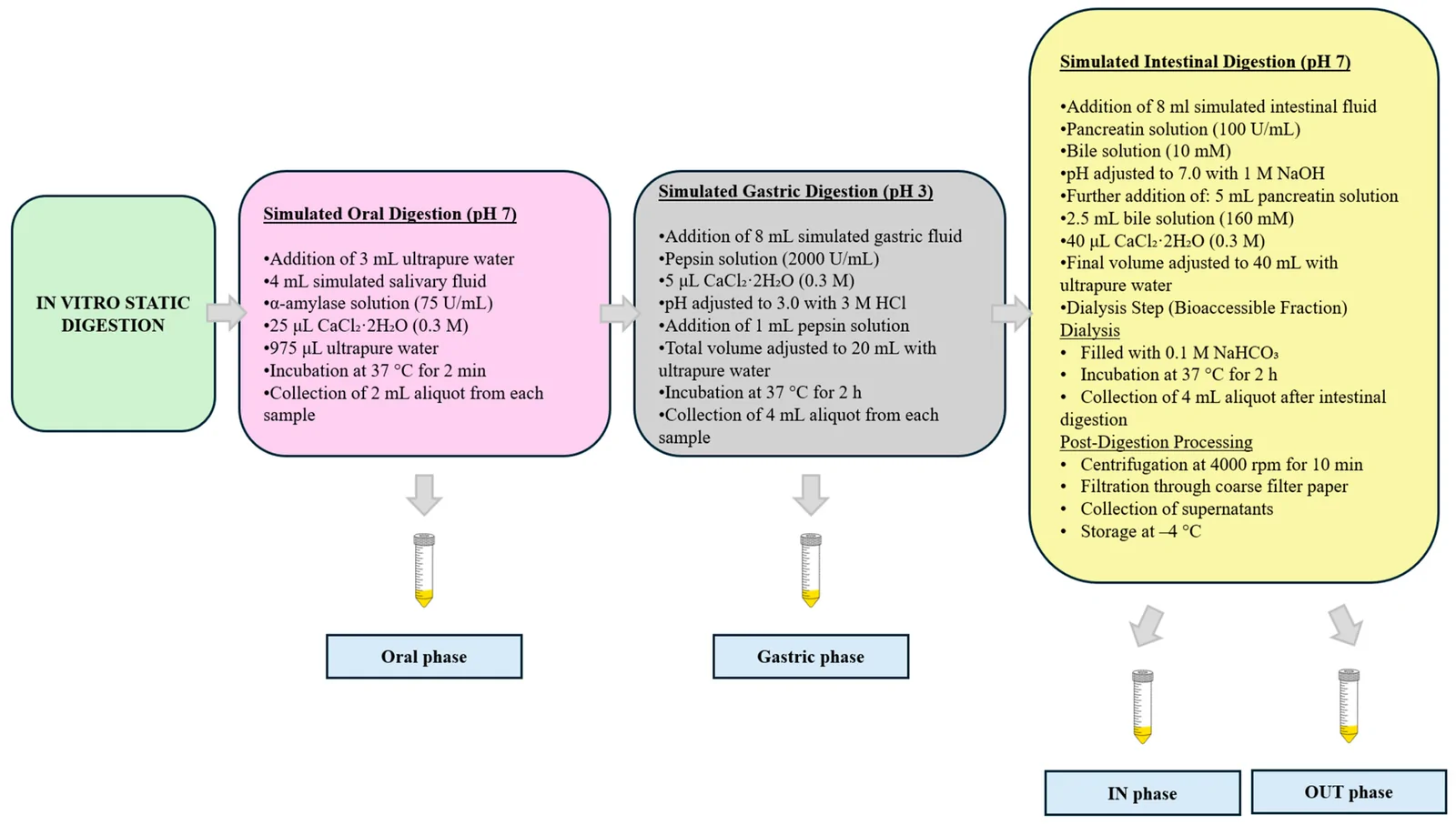
Schematic representation of the experimental workflow for the in vitro INFOGEST static gastrointestinal digestion protocol.

An in vitro static gastrointestinal digestion model based on the standardized INFOGEST protocol was employed to simulate the oral, gastric, and intestinal phases under physiologically relevant conditions. The complete experimental workflow, including the sequential digestion stages and the main processing conditions applied in each phase, is schematically presented in Figure 1. Following the intestinal phase, the digesta was separated into dialyzable and non-dialyzable fractions. The dialyzable fraction was designated as the IN phase and considered an in vitro proxy for the fraction potentially accessible for subsequent intestinal absorption, whereas the remaining non-dialyzable fraction was designated as the OUT phase. The bioaccessibility index (BI) was calculated as the percentage of the concentration of the target constituent recovered in the dialyzable intestinal fraction (IN phase) relative to its concentration in the corresponding undigested sample. Accordingly, the calculated values represent the proportion of the initial constituent that became potentially accessible for absorption under the applied in vitro digestion conditions and should therefore be interpreted as estimates of in vitro bioaccessibility rather than as direct evidence of physiological intestinal absorption or in vivo bioavailability. The model was applied to evaluate the in vitro bioaccessibility of minerals, amino acids, fatty acids, phenolic compounds, and antioxidant capacity [25].

### 2.5. Mineral Analysis

Mineral composition was determined following microwave-assisted acid digestion using atomic absorption spectrophotometry. Major (Ca, K, Na, Mg, and P) and trace elements (Fe, Zn, Cu, and Mn) were quantified using certified external standards, and the results were expressed as mg/100 g sample [26].

### 2.6. Total Phenolic Content (TPC)

Total phenolic content was determined using the Folin–Ciocalteu colorimetric assay, with gallic acid as the calibration standard (0.1–1 mg/mL; y = 9.6601x + 0.0172; R^2^ = 0.9997). Results were expressed as mg gallic acid equivalents (GAE) per 100 g sample [27].

### 2.7. Antioxidant Capacity Assays

#### 2.7.1. Cupric Ion Reducing Antioxidant Capacity (CUPRAC) Assay

CUPRAC activity was determined according to the reduction of the Cu(II)-neocuproine complex, using Trolox (6-hydroxy-2,5,7,8-tetramethylchroman-2-carboxylic acid, Sigma-Aldrich Corporation, Shanghai, China) for calibration (0.04–0.8 mg/mL; y = 1.4568x − 0.0362; R^2^ = 0.9966). Antioxidant activity was expressed as μmol Trolox equivalents (TE) per gram of sample [28].

#### 2.7.2. ABTS Radical Scavenging Activity

ABTS radical scavenging activity was evaluated by measuring the reduction of the ABTS•^+^ radical cation after reaction with sample extracts. Antioxidant capacity was quantified against a Trolox calibration curve (0.01–0.2 mg/mL; y = 3.4176x + 0.0504; R^2^ = 0.9991) and expressed as mg TE g^−1^ [29].

#### 2.7.3. Ferric Reducing Antioxidant Power (FRAP)

FRAP activity was assessed by measuring the reduction of the Fe^3+^–TPTZ complex to its ferrous form under acidic conditions using a calibration curve (1 mmol/L; y = 5.3035x + 0.0279; R^2^ = 0.9965). Results were expressed as μmol Fe^2+^ equivalents per gram of sample [30].

### 2.8. Amino Acid Analysis

Amino acid composition was determined following acid and alkaline hydrolysis using pre-column derivatization and ultra-fast liquid chromatography (UFLC). Amino acid derivatives were quantified using a Shimadzu HPLC system (Kyoto, Japan) equipped with a fluorescence detector. Separation was performed on an Inertsil ODS-80A (GL Sciences, Tokyo, Japan) reversed-phase column (150 × 4.6 mm i.d., 5 μm) maintained at 40 °C, with a flow rate of 1.0 mL/min. Fluorescence detection was performed at excitation/emission wavelengths of 490/530 nm for NBD-PyNCS derivatives and 460/540 nm for DBD-PyNCS derivatives. Seventeen different amino acids were quantified based on external calibration using authentic standards [31].

### 2.9. Phenolic Compound Analysis

Individual phenolic compounds were identified and quantified by high-performance liquid chromatography coupled with diode-array detection (HPLC-DAD). Quantitative analysis was carried out using an HPLC system equipped with an LC-20AD pump, an SPD-M20A diode array detector (DAD), and a SIL-20AHT autosampler (Shimadzu Corporation, Kyoto, Japan). The separation was performed using a C18 Intersil^®^ ODS column (250 × 4.6 mm, 5 μm) at 40 °C and a flow rate of 1 mL/min. A gradient elution mode was applied using 0.1% acetic acid in water (A) and 0.1% acetic acid in acetonitrile (B), with the proportion of B programmed as follows: 10% (0–2 min), 30% (2–27 min), 90% (27–50 min), and 100% (50–60 min). The mobile phase composition was subsequently returned to the initial condition at 63 min. Compound identification was achieved by comparing retention times and UV spectra with reference standards, while quantification was performed using external calibration curves [32].

### 2.10. Fatty Acid Profile Analysis

The fatty acid composition of tahini, pea protein powder, coconut milk powder, spray-dried powder, and its intestinal digesta obtained after in vitro gastrointestinal digestion was determined with minor modifications, following the procedure reported by Tüfekci and Karataş [33]. Briefly, lipid fractions of the solid samples were extracted with petroleum ether (boiling ramge, 40–60 °C) using a Soxhlet extractor at 55 °C for 15 h. The solvent was removed under vacuum using a rotary evaporator (Heidolph Instruments GmbH & Co. KG, Schwabach, Germany) at 40 °C for approximately 15 min. For fatty acid methyl ester (FAME) preparation, 0.1 g of the extracted lipid was dissolved in 7 mL n-heptane and vigorously mixed with 0.75 mL of 2 N methanolic KOH. Following centrifugation at 3075× *g* for 5 min, 1 mL of the upper phase was transferred to an autosampler vial. Fatty acids were analyzed using an Agilent 6890N gas chromatograph (Agilent Technologies, Waldbronn, Germany) equipped with a flame ionization detector and a DB-23 capillary column (60 m × 0.25 mm × 0.25 μm). The injector and detector temperatures were maintained at 250 and 260 °C, respectively, with nitrogen as the carrier gas at 1.4 mL/min. The injection volume was 0.2 μL with a split ratio of 1:40. The oven temperature was initially held at 130 °C for 1 min, increased to 170 °C at 6.5 °C/min, then to 215 °C at 2.75 °C/min and held for 15 min, followed by an increase to 230 °C at 15 °C/min and a final hold for 10 min. FAMEs were identified by comparison of their retention times with those of a Supelco F.A.M.E. 37 Mix (Bellefonte, PA, USA) standard.

### 2.11. Statistical Analysis

All analytical measurements were conducted in triplicate, and the results were expressed as mean values accompanied by their corresponding standard deviations. Statistical evaluation of the data was performed using the SPSS software package (Version 16.0, SPSS Inc., Chicago, IL, USA). Differences among sample groups were evaluated using one-way analysis of variance (ANOVA), followed by Tukey’s multiple comparison test. Statistical significance was set at *p* < 0.05.

## 3. Results and Discussion

### 3.1. Proximate Composition

The proximate composition of tahini, pea protein powder, coconut milk powder, and the developed spray-dried powder is presented in Table 1. Statistical comparison among the raw materials and the final formulation was conducted to assess how the incorporation of the individual ingredients contributed to the overall nutritional composition of the developed powder. Significant differences were observed between the samples, reflecting the distinct compositional characteristics of each raw material and their combined contribution to the nutritional profile of the final formulation. Tahini was characterized by its high lipid content (52.53%), together with considerable amounts of protein (21.84%) and dietary fiber (3.34%), confirming its nutritional value as a lipid- and protein-rich sesame product. These findings are consistent with the naturally oil-rich composition of sesame seeds and agree with previous reports describing tahini as an important source of plant proteins and unsaturated lipids [34]. Moreover, the physicochemical characteristics of the tahini complied with the quality specifications established by the Turkish Food Codex [6], demonstrating the suitability of the raw material for incorporation into spray-dried formulations. Similar compositional values have also been reported by Cevik et al. [35], although minor variations are expected due to differences in sesame cultivar, roasting conditions, and milling technology.

As expected, pea protein powder exhibited the highest protein concentration (81.30%) among all ingredients while containing relatively low amounts of lipids and carbohydrates. Such composition highlights its role as the principal protein source within the formulation and explains the marked increase in protein content observed in the final powder. The protein concentration obtained in the present study falls within the range previously reported for commercial pea protein ingredients [36], confirming its suitability for developing high-protein plant-based foods.

Coconut milk powder presented a contrasting composition dominated by lipids (50.02%) and carbohydrates (40.07%), whereas protein and fiber contents remained comparatively low. The elevated lipid fraction primarily originates from coconut-derived saturated triglycerides, while the carbohydrate fraction is associated with naturally occurring sugars and drying carrier agents commonly incorporated during powder production [37]. Its relatively low moisture content further reflects the effectiveness of spray-drying technology in producing microbiologically stable coconut-based powders [38].

The spray-dried powder successfully integrated the nutritional characteristics of all formulation components, resulting in a product containing 52.69% protein, 26.42% lipids, 8.28% crude fiber, 6.95% carbohydrates, and only 2.28% moisture. This balanced composition demonstrates that combining microfluidization with spray drying effectively produced a protein-enriched powder while maintaining moderate lipid levels and substantially increasing dietary fiber content. The low moisture level is particularly important because water availability is one of the primary factors governing physicochemical stability, lipid oxidation, microbial growth, and storage performance of powdered [11,39]. Simultaneously, the elevated protein and fiber contents substantially improve the nutritional quality of the formulation and enhance its potential as a functional ingredient for plant-based beverages, bakery products, nutritional supplements, and high-protein food systems [40]. Overall, these findings demonstrate that the selected formulation strategy successfully generated a nutritionally balanced powder while preserving the desirable compositional characteristics of its individual ingredients.

### 3.2. Color Characteristics

Tahini exhibited the lowest lightness (L*) value owing to its roasted sesame composition and naturally high lipid content. Roasting promotes non-enzymatic browning reactions and pigment formation, reducing light reflectance and producing the characteristic creamy-brown appearance of tahini [41]. In contrast, coconut milk powder showed the highest L* value, reflecting its naturally white appearance, whereas pea protein powder also displayed relatively high lightness. However, the spray-dried powder exhibited an intermediate L* value, indicating that incorporation of tahini reduced overall brightness while the lighter pea protein and coconut powders partially compensated for this effect. Such behaviour may reflect improved dispersion of lipid droplets within the continuous matrix following microfluidization, which could influence light scattering and contribute to the observed colour changes without necessarily indicating excessive thermal browning during spray drying. Similar optical behaviour has been reported for complex emulsion systems, where particle size distribution, refractive index differences, and microstructural organization largely determine light reflection and perceived brightness [38,42]. The redness parameter (a*) followed a similar trend (Table 1), with tahini exhibiting the highest positive value due to roasting-derived pigments. The intermediate a* value observed in the spray-dried powder indicates that the contribution of sesame pigments was moderated after homogenization with the lighter formulation components, producing a visually balanced product. This finding suggests that formulation effects may have played a greater role than thermal degradation in determining the final color characteristics. Regarding yellowness (b*) (Table 1), pea protein powder presented the highest value, followed by tahini, whereas coconut milk powder showed considerably lower yellowness. The final spray-dried powder again displayed intermediate values, indicating that pigment dilution and matrix formation influenced the optical response of the composite system. Besides pigment concentration, alterations in particle morphology generated during spray drying may also contribute to changes in yellowness by modifying light absorption and scattering behavior [43].

Although thermal processing may promote Maillard reactions capable of darkening powder products [40,42], the relatively high L* value together with moderate a* and b* values suggest that the selected spray-drying conditions successfully minimized excessive browning while preserving the characteristic appearance of the formulation. This observation agrees with previous reports demonstrating that optimization of inlet temperature, feed concentration, and residence time effectively limits undesirable color deterioration in spray-dried plant-based powders [38,40]. From an application perspective, the intermediate color profile obtained in the present study is advantageous because it broadens the potential use of the powder in different food formulations without causing pronounced color changes in the final product. Therefore, the combination of microfluidization and spray drying not only preserved the visual quality of the individual ingredients but also generated a homogeneous powder with desirable appearance and high technological suitability for functional food applications.

### 3.3. Mineral Content

The mineral compositions of tahini, pea protein powder, coconut milk powder, spray-dried powder, and the intestinal fraction obtained after simulated gastrointestinal digestion are summarized in Table 2. Significant differences between the samples indicate that mineral distribution was primarily determined by the intrinsic composition of the ingredients, whereas the subsequent changes observed after simulated digestion reflected the combined effects of matrix composition and the physicochemical conditions of the gastrointestinal model. Tahini was the predominant mineral source within the formulation, exhibiting remarkably high concentrations of phosphorus, potassium, calcium, and magnesium together with appreciable amounts of sodium, iron, zinc, copper, and manganese. This mineral-rich profile reflects the intrinsic nutritional value of sesame seeds, which are recognized as excellent dietary sources of macro- and trace elements owing to their naturally high ash content and dense seed matrix. Comparable mineral distributions have previously been reported for tahini and sesame products [44], although quantitative differences among studies are expected because of genotype, cultivation practices, environmental conditions, roasting intensity, and processing technology. Accordingly, the relatively high mineral content of the developed formulation was likely attributable primarily to the contribution of the tahini fraction, with limited influence from the processing conditions.

In contrast, coconut milk powder contained substantially lower concentrations of most minerals, with the exception of manganese, which was present at the highest concentration among the investigated raw materials. Potassium and sodium represented the major minerals in coconut powder, whereas calcium, phosphorus, magnesium, iron, zinc, and copper were comparatively limited. Such differences are likely related to the intrinsic botanical composition of coconut endosperm and the potential dilution effect associated with carbohydrate-rich carrier materials commonly used during spray drying. Previous investigations have similarly demonstrated that mineral composition in coconut-derived powders is highly dependent on extraction conditions, particle size, and drying methodology, highlighting the influence of technological variables on mineral retention [45]. Pea protein powder exhibited moderate mineral concentrations, with potassium representing the dominant element, followed by sodium, phosphorus, magnesium, and calcium. Although its mineral contribution was lower than that of tahini, pea protein provided additional amounts of iron, manganese, zinc, and copper while serving as the principal protein source of the formulation. Differences from previously reported mineral values may be associated with pea cultivar, protein isolation technology, and agricultural conditions [46].

The spray-dried powder retained measurable amounts of all investigated minerals after processing with, phosphorus remaining the predominant mineral, followed by calcium, potassium, and magnesium, whereas trace minerals such as iron, zinc, manganese, and copper were also detected. The presence of these minerals in the final powder is consistent with the generally high thermal stability of inorganic minerals during food processing. However, because the present study did not include separate processing controls, the extent to which microfluidization or spray drying individually influenced mineral recovery cannot be established. The minor differences observed between the raw ingredients and the spray-dried formulation may instead reflect differences in matrix composition, mineral distribution, or analytical extractability rather than mineral destruction. Similar stability of mineral composition has been reported in other spray-dried food systems [47].

Following simulated gastrointestinal digestion, the concentrations of all minerals detected in the intestinal fraction decreased considerably, indicating that only a proportional of the minerals present in the undigested formulation was transferred to the potentially bioaccessible intestinal fraction. This decrease should not be interpreted as direct mineral loss; rather, it likely reflects incomplete release, solubilization, or transfer of minerals from the food matrix under the simulated gastrointestinal conditions. Mineral bioaccessibility is primarily governed by solubilization processes occurring under dynamic gastrointestinal conditions and is strongly influenced by interactions between minerals and surrounding macromolecules [48,49]. Proteins, dietary fibre, and particularly phytates may chelate divalent and trivalent cations, thereby limiting their incorporation into soluble intestinal fractions [50,51]. The imposed changes in pH, digestive enzymes, bile components, and subsequent phase separation may have further affected mineral solubilization and recovery. Thus, the lower mineral concentrations measured in the intestinal fraction primarily indicate that only a fraction of the minerals became accessible under the specific conditions of the digestion model.

Interestingly, iron displayed the greatest bioaccessibility despite generally being considered one of the minerals most susceptible to inhibition by phytates and polyphenols. Rather than interpreting this finding as evidence that the formulation specifically enhanced iron bioavailability, it may indicate that iron was relatively more readily released or remained soluble under the applied digestion conditions compared with the other minerals. The relatively high iron bioaccessibility may be associated with differences in mineral–matrix interactions and the physicochemical behavior of iron during digestion.

Overall, the mineral profile of the developed powder primarily reflected the complementary contributions of tahini, pea protein, and coconut milk powder, with tahini representing the major mineral source. The detection of all investigated minerals after spray drying indicates that the final powder retained a substantial mineral complement, while the lower concentrations observed after simulated digestion demonstrate that mineral release into the potentially bioaccessible intestinal fraction was incomplete. The observed bioaccessibility therefore reflects the combined influence of ingredient composition, matrix characteristics, processing history, and gastrointestinal conditions rather than a demonstrated effect of microfluidization or spray drying individually. These findings support the developed powder as a mineral-containing plant-based ingredient, while cellular uptake and in vivo studies would be required to establish its physiological nutritional relevance.

### 3.4. Fatty Acid Composition

The fatty acid profiles of tahini, pea protein powder, coconut milk powder, spray-dried powder, and its intestinal fraction following in vitro gastrointestinal digestion are presented in Table 3. The differences among the raw ingredients reflected their distinct lipid compositions, whereas the changes observed after digestion provided insight into the differential release and recovery of individual fatty acids under the simulated gastrointestinal conditions. Tahini exhibited a lipid profile dominated by unsaturated fatty acids, with linoleic acid (43.73%) and oleic acid (42.53%) accounting for the majority of total fatty acids, followed by palmitic (10.83%) and stearic acids (5.35%). Minor amounts of arachidic, linolenic, palmitoleic, lauric, and behenic acids were also detected. The predominance of oleic and linoleic acids is consistent with the characteristic lipid profile of sesame and underpins the nutritional relevance of tahini as a source of unsaturated fatty acids [52]. Similar distributions have been reported for commercial tahini and sesame-based products [53,54], while quantitative variation among studies may reflect differences in genotype, geographical origin, cultivation conditions, roasting intensity, and processing technology [44,52,53,54,55].

Coconut milk powder exhibited a markedly different profile, being dominated by lauric acid (74.28%), followed by myristic and palmitic acids. This distribution agrees with the established fatty acid composition of coconut-derived products, in which lauric acid is typically the predominant fatty acid [56]. Thus, coconut milk powder introduced a distinct medium-chain saturated fatty acid fraction into the formulation, complementing the predominantly unsaturated lipid profile of tahini. Medium-chain fatty acids have distinct digestive and metabolic characteristics compared with long-chain fatty acids, including relatively rapid absorption and hepatic utilization [57]. Moreover, pea protein powder, although primarily included as a protein source, contained a measurable residual lipid fraction dominated by linoleic acid (48.45%), followed by oleic, palmitic, linolenic, and stearic acids. Its lipid contribution was therefore characterized predominantly by unsaturated fatty acids, further contributing to the overall diversity of the formulation. The observed profile was broadly consistent with previously reported fatty acid distributions for pea-derived ingredients [58], with quantitative differences potentially arising from cultivar and processing-related variation.

The spray-dried powder exhibited an intermediate fatty acid profile reflecting the combined lipid contributions of the three ingredients. Oleic (37.85%) and linoleic acids (34.65%) remained the predominant unsaturated fatty acids, whereas lauric (12.43%), palmitic (6.64%), myristic (4.76%), and stearic acids (2.23%) represented the major saturated components. The resulting profile therefore represented a transition between the unsaturated fatty acid-rich tahini/pea protein fractions and the lauric acid-rich coconut component. The persistence of the major fatty acids after processing indicates that the characteristic lipid profile was maintained in the resulting powder. However, because the study did not include a non-microfluidized or non-dried processing control, these data do not allow the effects of microfluidization or spray drying on fatty acid stability to be isolated. The observed fatty acid profile may be partly related to the relative chemical stability of fatty acids under the controlled thermal conditions applied during spray drying.

Following simulated gastrointestinal digestion, all major fatty acids remained detectable in the intestinal fraction, although their concentrations were considerably lower than those measured in the undigested powder. This pattern indicates that only a fraction of the lipid-associated fatty acids was recovered in the intestinal phase under the applied digestion conditions. Such differences in recovery are expected because lipid digestion involves sequential hydrolysis, dispersion, and incorporation of digestion products into mixed micelles, processes that are influenced by the structure of the lipid matrix, accessibility of lipase to the lipid–water interface, triacylglycerol composition, and micelle formation [59]. Accordingly, the lower concentrations in the intestinal fraction should primarily be interpreted as differences in digestive release and phase recovery rather than as direct evidence of fatty acid degradation.

The BI further demonstrated that digestive behavior differed considerably among individual fatty acids. Saturated fatty acids, particularly palmitic, caprylic, lauric, myristic, and stearic acids, exhibited comparatively higher bioaccessibility, whereas oleic, linoleic, and especially linolenic acids showed lower values. Differences in chain length and degree of unsaturation can influence lipase-mediated hydrolysis, solubilization, and incorporation into mixed micelles [60]. In particular, medium-chain fatty acids generally exhibit greater aqueous solubility and can be transferred more readily into the aqueous digestive phase, whereas long-chain fatty acids depend more strongly on bile-mediated emulsification and mixed-micelle formation. The lower bioaccessibility of highly unsaturated long-chain fatty acids may therefore reflect differences in their physicochemical behavior during digestion rather than a simple relationship with their initial abundance in the powder. These observations are consistent with previous reports showing that fatty acid bioaccessibility is governed by molecular characteristics as well as matrix-dependent digestive processes [60,61,62,63]. Overall, the developed powder combined the contrasting lipid characteristics of tahini, pea protein, and coconut milk powder, resulting in a fatty acid profile containing both predominantly unsaturated long-chain fatty acids and substantial medium-chain saturated fatty acids. The major fatty acids remained detectable after spray drying, indicating that the characteristic lipid composition of the formulation was maintained in the final powder, although the present design does not permit attribution of this observation to microfluidization or spray drying individually. Following simulated gastrointestinal digestion, fatty acid recovery varied substantially according to molecular structure, with relatively greater bioaccessibility observed for several saturated fatty acids. These findings demonstrate that both ingredient composition and fatty acid molecular characteristics contribute to the gastrointestinal behavior of the developed formulation, while the observed in vitro bioaccessibility should be interpreted as an estimate of digestive-phase recovery rather than direct evidence of intestinal absorption or physiological benefit.

### 3.5. Amino Acid Composition

The amino acid profiles of tahini, pea protein powder, coconut milk powder, the spray-dried formulation, and IN phase obtained after simulated gastrointestinal digestion are presented in Table 4. The differences among the raw ingredients reflected their distinct protein compositions, whereas the changes following digestion provided information on the differential recovery of amino acids in the potentially bioaccessible intestinal fraction. Tahini exhibited relatively high concentrations of L-glutamic acid, L-aspartic acid, L-threonine, L-phenylalanine, and L-tryptophan, consistent with the characteristic amino acid composition of sesame proteins [54]. This profile was broadly comparable with previously reported amino acid distributions for sesame-based products [64], although quantitative differences may arise from genotype, environmental conditions, roasting intensity, and analytical methodology [65,66].

Pea protein powder contained the highest concentrations of most indispensable amino acids, particularly L-lysine, L-leucine, L-isoleucine, L-valine, and L-threonine, reflecting its high protein purity and nutritional quality. This lysine-rich profile is characteristic of legume proteins and complements the comparatively different amino acid distribution of sesame proteins [67,68]. Thus, the incorporation of pea protein contributed substantially to the indispensable amino acid content of the formulation. However, the present results demonstrate compositional complementarity rather than a statistically verified improvement in overall protein quality. In contrast, coconut milk powder contained considerably lower concentrations of both indispensable and dispensable amino acids, consistent with its relatively low protein contribution. Accordingly, its contribution to the amino acid profile of the final formulation was limited compared with tahini and pea protein, while its primary role in the formulation was related to providing complementary solids and compositional characteristics [69].

The spray-dried powder contained all major amino acids detected in the individual ingredients, and its overall amino acid profile reflected the combined contributions of tahini, coconut milk powder, and pea protein powder. The presence of these amino acids after microfluidization and spray drying indicates that the overall amino acid profile was largely preserved during processing. However, as separate processing controls were not included, the individual contributions of microfluidization and spray drying to these changes could not be determined. The relatively short thermal exposure during spray drying may have contributed to the preservation of the amino acid profile, although the present results do not allow the effects of the individual processing steps to be separated.

Following simulated gastrointestinal digestion, all major amino acids remained detectable in the intestinal fraction, demonstrating that amino acid-derived compounds were present in the digestion products. However, their concentrations were generally lower than those measured in the undigested powder, suggesting that only a proportion of the protein-derived components were recovered in the soluble intestinal fraction. This decrease may be associated with the extent of protein hydrolysis and the subsequent formation and partitioning of peptides and amino acids during simulated gastrointestinal digestion. Thus, the lower concentrations observed in the intestinal fraction may reflect differences in the release and distribution of protein-derived compounds during digestion rather than necessarily indicating their complete degradation.

The bioaccessibility indices showed considerable variation among individual amino acids. These differences may reflect not only the intrinsic characteristics of individual amino acids but also their occurrence within different protein structures and the interactions of proteins with other matrix components. Phenolic compounds, dietary fiber, and polysaccharides can interact with proteins and potentially influence enzyme accessibility and protein hydrolysis in complex plant-based matrices [20,70,71,72]. Accordingly, the observed differences in amino acid bioaccessibility cannot be attributed solely to the intrinsic properties of individual amino acids; they may also reflect protein structure, matrix interactions, processing history, and the physicochemical conditions imposed during simulated digestion [30].

Among the indispensable amino acids, L-lysine exhibited relatively high bioaccessibility. Given the susceptibility of lysine to processing-induced reactions such as Maillard-type interactions, its substantial recovery in the intestinal fraction indicates that a considerable proportion remained detectable under the applied digestion conditions; however, this finding does not demonstrate the absence of chemical modification or preservation of its nutritional quality. Likewise, the high bioaccessibility of branched-chain amino acids, including leucine, isoleucine, and valine, indicates their relatively high recovery in the intestinal fraction and may be nutritionally relevant given their established roles in protein metabolism, including muscle protein synthesis and tissue maintenance [73]. These observations are relevant from a compositional perspective, but they should not be interpreted as evidence of enhanced intestinal absorption or physiological effects because the present study assessed in vitro bioaccessibility rather than post-absorptive utilization.

Overall, the amino acid profile of the developed powder reflected the complementary contributions of sesame- and pea-derived proteins, with pea protein providing a particularly important contribution of lysine and other indispensable amino acids. The major amino acids remained measurable after processing and simulated gastrointestinal digestion, although their recovery in the intestinal fraction varied among individual amino acids. These findings indicate that the formulation retained a broad amino acid profile and that its digestive behavior was influenced by both protein composition and matrix-dependent processes. However, the present experimental design does not permit conclusions regarding protein quality, intestinal absorption, or physiological utilization; such assessments would require complementary approaches such as amino acid scoring, DIAAS/PDCAAS evaluation, cellular uptake models, or in vivo studies.

### 3.6. TPC and Antioxidant Activities

The TPC and antioxidant activities of tahini, pea protein powder, coconut milk powder, the spray-dried formulation, and their corresponding digested fractions are summarized in Table 5. Significant differences were observed between the samples, primarily reflecting the distinct phytochemical compositions of the individual ingredients and their relative contributions to the formulated matrix. Tahini exhibited the highest TPC among the raw materials, confirming sesame as an important dietary source of phenolic antioxidants. Sesame lignans, together with phenolic acids and flavonoids naturally present in the seed coat and cotyledon, are largely responsible for its elevated antioxidant capacity [35,74]. However, the spray-dried formulation exhibited a substantially higher TPC (53.47 mg GAE/100 g) than tahini (24.77 mg GAE/100 g), suggesting that the phenolic content of the final powder was influenced by the combined presence of the three ingredients and the processing conditions.

Pea protein powder also contributed to appreciable amounts of phenolic compounds, although its TPC and antioxidant activities were considerably lower than those of tahini, coconut milk powder, and the spray-dried formulation. Coconut milk powder exhibited intermediate TPC and antioxidant values among the raw ingredients. Thus, although tahini represented the richest individual source of phenolics among the three raw materials, the higher TPC observed in the spray-dried formulation suggests that the final matrix enhanced the overall measurable phenolic content relative to the individual ingredients. This effect may be attributable, at least in part, to the additive contribution of the different components and to changes in phenolic extractability induced by matrix formation and processing.

The antioxidant results broadly followed the overall phenolic pattern, although the relative ranking varied among assays. The spray-dried powder exhibited the highest ABTS (40.67 mg TE/g) and FRAP (53.80 μmol Fe^2+^/g) activities, whereas tahini showed the highest CUPRAC activity (41.30 μmol TE/g). These differences indicate that antioxidant responses were assay-dependent and cannot be explained solely by TPC. Antioxidant activity may additionally be influenced by the qualitative composition of phenolic compounds, antioxidant peptides, tocopherols, and other redox-active constituents. Thus, the antioxidant capacity of the formulation likely reflected the combined contribution of multiple bioactive constituents rather than a direct proportional relationship with total phenolic content.

Simulated gastrointestinal digestion markedly altered TPC. TPC decreased sharply during the oral phase, followed by a partial recovery during the gastric and intestinal stages. However, TPC remained substantially below the level of the undigested powder throughout digestion. The progressive recovery observed in the later phases may be associated with matrix disruption, pH changes, enzymatic hydrolysis, and the release of phenolic compounds previously associated with proteins or other macromolecules [75]. Accordingly, changes in TPC across the digestion phases are more appropriately interpreted as differences in phenolic extractability, release, and phase distribution rather than changes in the absolute amount of phenolic compounds.

Antioxidant activities showed a comparable but not identical response to digestion. All three assays exhibited a marked decrease during the oral phase, followed by partial recovery during the gastric and intestinal stages. Antioxidant activities in the OUT phase were consistently higher than those in the IN phase, indicating that the antioxidant capacity measured in the non-dialyzable fraction exceeded that recovered in the potentially bioaccessible intestinal fraction. Overall, digestion influenced the measured antioxidant activities, although the magnitude and direction of these changes varied depending on the assay employed.

The consistently lower antioxidant activities after digestion, despite partial recovery of TPC in the later phases, further demonstrate that antioxidant capacity was not determined by TPC alone. Phenolic release may increase their analytical extractability without necessarily preserving their original antioxidant activity, while changes in molecular structure, oxidation, interactions with digestive components, and the formation or release of other antioxidant compounds may alter the response of individual assay [76,77]. The higher antioxidant activity observed in the OUT phase than in the IN phase across all three assays is particularly noteworthy and suggests that a considerable proportion of the antioxidant-active compounds remained associated with the non-dialyzable matrix rather than being transferred to the potentially bioaccessible intestinal fraction.

The calculated bioaccessibility index for TPC was 44.32%, indicating that approximately 44% of the TPC measured in the undigested spray-dried powder was recovered in the IN phase. Thus, despite the substantial changes in phenolic recovery and antioxidant activity during simulated digestion, a measurable proportion of the initial phenolic content remained present in the potentially bioaccessible intestinal fraction under the applied in vitro conditions.

Overall, the spray-dried formulation exhibited higher TPC and generally greater antioxidant responses than the individual raw ingredients, although the antioxidant profile varied according to the assay employed. Simulated gastrointestinal digestion substantially modified both phenolic recovery and antioxidant activity, with partial recovery of TPC and antioxidant capacity during the gastric and intestinal stages, while values generally remained below those of the undigested powder. The higher antioxidant responses in the OUT phase compared with the IN phase suggest that a substantial proportion of the measured antioxidant capacity was retained in the non-dialyzable fraction. The observed changes therefore may reflect the combined effects of ingredient composition, matrix-related interactions, compound-specific stability, and gastrointestinal conditions, rather than providing direct evidence of a protective effect of microfluidization or synergistic interactions among the ingredients. Overall, the results demonstrate measurable phenolic bioaccessibility and antioxidant activity under the applied in vitro conditions, while further studies are needed to establish the extent to which these findings translate to intestinal uptake and physiological effects.

### 3.7. Phenolic Acid Profile

The phenolic compound profiles of tahini, pea protein powder, coconut milk powder, spray-dried powder, and the IN phase of the spray-dried powder are presented in Table 6. Significant differences between the samples were observed for all identified phenolic compounds, demonstrating marked differences in the phenolic composition of the individual ingredients and the resulting formulation.

Tahini was characterized by a phenolic profile dominated by gallic acid (119.34 µg/g), whereas the other identified phenolic compounds occurred at considerably lower concentrations. In comparison, pea protein powder exhibited a broader phenolic profile, with benzoic acid reaching the highest concentration (879.68 µg/g), accompanied by relatively high levels of quercetin, syringic acid, ferulic acid, and (+)-catechin. Coconut milk powder contributed additional phenolic constituents, particularly ferulic acid (70.34 µg/g) and benzoic acid (64.38 µg/g). Collectively, these differences highlight the distinct phytochemical contributions of the individual ingredients and suggest that the phenolic composition of the final formulation was influenced by the complementary profiles of its constituent materials. The spray-dried powder exhibited a diverse phenolic profile, with benzoic acid (137.84 µg/g) and gallic acid (68.35 µg/g) as the predominant identified compounds, followed by quercetin and syringic acid. The detection of these phenolic constituents in the final powder indicates that several major compounds remained analytically measurable following processing. Nevertheless, in the absence of an unprocessed or non-microfluidized control, the present data do not permit the specific contribution of microfluidization or spray drying to the retention of individual phenolic compounds to be established. The phenolic profile observed in the final powder may therefore reflect the initial phytochemical composition of the ingredients, differences in the stability of individual phenolic compounds during processing, and variations in their extractability within the formulated matrix.

Simulated gastrointestinal digestion substantially altered the phenolic profile. Most compounds showed lower concentrations in the IN phase, with benzoic acid (39.82 µg/g), gallic acid (26.73 µg/g), quercetin (8.92 µg/g), and syringic acid (7.24 µg/g) remaining the major detected constituents. The lower recovery in the IN phase should not be interpreted solely as phenolic degradation, because it may also reflect incomplete release from the matrix, binding to proteins or other macromolecules, chemical transformation, and limited transfer into the dialyzable fraction [78,79]. Changes in pH, digestive enzymes, bile components, and phase partitioning may further influence the stability and recovery of individual phenolics during digestion [76,79].

The bioaccessibility indices demonstrated pronounced compound-specific differences. (+)-Catechin showed the highest bioaccessibility (53.59%), followed by quercetin (49.47%), syringic acid (40.33%), gallic acid (39.11%), and apigenin (36.30%), whereas ferulic acid, caffeic acid, and vanillic acid exhibited comparatively lower values. This variability suggests that the bioaccessibility of individual phenolic compounds was influenced by their distinct chemical characteristics and interactions with the food matrix. Differences in molecular structure, polarity, pH-dependent stability, and susceptibility to transformation may contribute to these distinct digestive behaviors [76,80].

Overall, the spray-dried powder exhibited a diverse and measurable phenolic profile, although the present experimental design does not allow the specific effects of microfluidization or spray drying on the individual phenolic compounds to be established. More importantly, simulated gastrointestinal digestion altered the recovery of individual phenolic compounds, resulting in substantial differences in their bioaccessibility indices. These findings are consistent with previous observations that gastrointestinal digestion can alter both the recovery and bioaccessibility of individual phenolics, with the extent of these changes depending on their chemical characteristics and interactions with the food matrix [20,76,78]. Thus, the present results demonstrate compound-specific in vitro phenolic bioaccessibility under the applied digestion conditions, while the extent to which these findings reflect intestinal absorption or physiological availability remains to be established.

## 4. Conclusions

This study characterized a tahini-based powder formulated with tahini, pea protein, and coconut milk powder in terms of its physicochemical properties, nutritional composition, phenolic profile, and in vitro bioaccessibility following simulated gastrointestinal digestion. The formulation combined the distinct nutritional and phytochemical characteristics of its constituent ingredients, resulting in a product with a diverse composition of proteins, amino acids, fatty acids, minerals, and phenolic compounds. Tahini contributed substantially to the lipid, mineral, and phenolic characteristics of the formulation, whereas pea protein contributed particularly to its protein and amino acid profile. The amino acid composition of the spray-dried powder was characterized by relatively high levels of glutamic acid, aspartic acid, leucine, and lysine, while oleic and linoleic acids were the predominant fatty acids. The formulation also contained measurable levels of nutritionally relevant minerals, with phosphorus, potassium, calcium, and magnesium representing the major mineral constituents. Following simulated gastrointestinal digestion, the bioaccessibility index of individual minerals varied considerably, indicating component-specific differences in their in vitro bioaccessibility. The major amino acids remained detectable in the IN phase, although their concentrations generally decreased compared with the undigested powder. Similarly, the major fatty acids remained measurable in the intestinal phase, with their bioaccessibility index (%) varying among individual fatty acids. Phenolic compounds also exhibited substantial differences in bioaccessibility index (%), with (+)-catechin showing the highest value among the identified phenolics. Antioxidant capacity varied across the digestion phases and according to the assay employed, with antioxidant responses generally higher in the OUT fraction than in the potentially bioaccessible IN fraction. Collectively, these findings demonstrate that the gastrointestinal behavior and in vitro bioaccessibility of the investigated constituents were component-dependent, with measurable amounts of nutritional and bioactive compounds remaining in the potentially bioaccessible intestinal fraction. The observed differences in bioaccessibility index may reflect the intrinsic characteristics of individual compounds, the composition and physicochemical properties of the food matrix, and the conditions encountered during gastrointestinal digestion. However, because the present study did not include non-microfluidized or non-spray-dried controls, the specific effects of microfluidization and spray drying on the bioaccessibility of individual constituents cannot be established. Accordingly, the present findings should not be interpreted as evidence of a specific protective, enhancing, or synergistic effect of these processing steps. Overall, the developed powder represents a nutritionally and phytochemically diverse plant-based formulation exhibiting measurable in vitro bioaccessibility following simulated gastrointestinal digestion. The findings provide a basis for further investigation of the formulation and its gastrointestinal behavior. Future studies incorporating appropriate processing controls, storage stability, reconstitution and dispersibility, sensory evaluation, and cellular or in vivo models are warranted to clarify its technological performance and to determine whether the observed in vitro bioaccessibility is associated with intestinal uptake or physiological effects.

## Figures and Tables

**Table 1:** Chemical composition and colorimetric properties of tahini, pea protein powder, coconut milk powder, and functional tahini powder.

Samples	Moisture (%)	Ash (%)	Crude Fiber (%)	Protein (%)	Lipids (%)	Carbohydrate (%)	pH	L*	a*	b*
Tahini	1.48 ± 0.04 ^d^	2.48 ± 0.03 ^c^	3.34 ± 0.09 ^b^	21.84 ± 0.28 ^c^	52.53 ± 0.08 ^a^	18.33 ± 0.03 ^b^	6.55 ± 0.07 ^d^	56.93 ± 0.12 ^d^	4.01 ± 0.05 ^a^	22.88 ± 0.07 ^b^
Pea protein powder	4.89 ± 0.18 ^a^	0.62 ± 0.05 ^d^	0.85 ± 0.18 ^c^	81.30 ± 0.36 ^a^	8.55 ± 0.23 ^d^	3.79 ± 0.22 ^d^	6.73 ± 0.04 ^c^	84.83 ± 1.13 ^b^	0.11 ± 0.02 ^c^	24.65 ± 0.35 ^a^
Coconut milk powder	4.67 ± 0.14 ^b^	2.65 ± 0.18 ^b^	0.27 ± 0.04 ^d^	2.02 ± 0.06 ^d^	50.02 ± 0.10 ^b^	40.07 ± 0.29 ^a^	6.84 ± 0.12 ^b^	93.24 ± 0.02 ^a^	−0.83 ± 0.07 ^d^	11.42 ± 0.08 ^d^
Spray dried powder	2.28 ± 0.13 ^c^	3.38 ± 0.03 ^a^	8.28 ± 0.04 ^a^	52.69 ± 0.58 ^b^	26.42 ± 0.05 ^c^	6.95 ± 0.01 ^c^	7.06 ± 0.07 ^a^	81.46 ± 0.32 ^c^	2.22 ± 0.06 ^b^	19.84 ± 0.26 ^c^

^a–d^: Different lowercase letters within the same column indicate statistically significant differences among samples, as determined by Tukey’s multiple comparison test (*p* < 0.05). Means sharing the same letter are not significantly different.

**Table 2:** Mineral content (mg/100 g) of tahini, pea protein powder, coconut milk powder, and plant based functional tahini powder.

Mineral Type	Tahini (Undigested Sample)	Coconut Milk Powder (Undigested Sample)	Pea Protein Powder (Undigested Sample)	Spray Dried Powder (Undigested Sample)	Spray Dried Powder (Intestinal Phase)	BI (%)
Calcium (Ca)	4264.65 ± 105.35 ^a^	43.54 ± 0.06 ^e^	113.74 ± 0.07 ^d^	2362.64 ± 94.52 ^b^	953.63 ± 34.62 ^c^	40.36
Potassium (K)	5446.52 ± 87.35 ^a^	1374.72 ± 13.64 ^c^	463.72 ± 13.63 ^e^	3252.67 ± 103.63 ^b^	942.63 ± 16.83 ^d^	28.98
Sodium (Na)	746.64 ± 17.46 ^a^	482.47 ± 2.57 ^b^	251.12 ± 3.63 ^d^	414.56 ± 12.25 ^c^	133.48 ± 9.46 ^e^	32.20
Magnesium (Mg)	2957.36 ± 41.97 ^a^	78.46 ± 0.04 ^e^	152.93 ± 0.09 ^d^	1156.63 ± 95.73 ^b^	479.82 ± 2.24 ^c^	41.48
Phosphorus (P)	9748.48 ± 111.42 ^a^	226.71 ± 1.28 ^d^	179.38 ± 1.25 ^e^	6653.72 ± 167.66 ^b^	1567.74 ± 101.54 ^c^	23.56
Iron (Fe)	84.64 ± 0.93 ^a^	5.19 ± 0.02 ^e^	8.27 ± 0.02 ^d^	57.29 ± 0.13 ^b^	25.35 ± 1.78 ^c^	44.25
Zinc (Zn)	73.63 ± 0.69 ^a^	2.83 ± 0.02 ^d^	2.78 ± 0.01 ^e^	28.93 ± 0.08 ^b^	11.34 ± 0.09 ^c^	39.20
Copper (Cu)	13.64 ± 0.86 ^a^	1.27 ± 0.01 ^d^	0.46 ± 0.01 ^e^	5.23 ± 0.05 ^b^	2.13 ± 0.01 ^c^	40.73
Manganese (Mn)	14.91 ± 0.74 ^b^	83.62 ± 0.98 ^a^	12.63 ± 0.03 ^c^	7.95 ± 0.03 ^d^	2.93 ± 0.01 ^e^	36.86

^a–e^: Different lowercase letters within the same row indicate statistically significant differences among samples, as determined by Tukey’s multiple comparison test (*p* < 0.05). Means sharing the same letter are not significantly different. BI: Bioaccessibility index.

**Table 3:** Fatty acid composition of tahini, coconut milk powder, and pea protein powder as well as undigested and intestinal phase of protein-rich functional tahini powder.

Fatty Acid Content (%)	Tahini (Undigested Sample)	Coconut Milk Powder (Undigested Sample)	Pea Protein Powder (Undigested Sample)	Spray Dried Powder (Undigested Sample)	Spray Dried Powder (Intestinal Phase)	BI (%)
Caproic acid (C6:0)	0.01 ± 0.00 ^b^	0.36 ± 0.00 ^a^	0.01 ± 0.00 ^b^	0.01 ± 0.00 ^b^	-	-
Caprylic acid (C8:0)	0.01 ± 0.00 ^d^	6.23 ± 0.02 ^a^	0.01 ± 0.00 ^d^	0.41 ± 0.01 ^b^	0.23 ± 0.01 ^c^	56.10
Capric acid (C10:0)	0.01 ± 0.00 ^a^	0.01 ± 0.00 ^a^	0.01 ± 0.00 ^a^	0.01 ± 0.00 ^a^	-	-
Lauric acid (C12:0)	0.04 ± 0.00 ^d^	74.28 ± 0.03 ^a^	0.01 ± 0.00 ^e^	12.43 ± 0.01 ^b^	6.84 ± 0.01 ^c^	55.03
Myristic acid (C14:0)	0.01 ± 0.00 ^d^	33.11 ± 0.04 ^a^	0.01 ± 0.00 ^d^	4.76 ± 0.01 ^b^	2.45 ± 0.01 ^c^	51.47
Palmitic acid (C16:0)	10.83 ± 0.00 ^c^	25.46 ± 0.04 ^a^	13.83 ± 0.01 ^b^	6.64 ± 0.01 ^d^	3.63 ± 0.01 ^e^	54.67
Palmitoleic acid (C16:1)	0.11 ± 0.03 ^a^	0.01 ± 0.00 ^b^	0.01 ± 0.00 ^b^	0.01 ± 0.00 ^b^	-	-
Stearic acid (C18:0)	5.35 ± 0.00 ^a^	4.62 ± 0.02 ^b^	3.69 ± 0.01 ^c^	2.23 ± 0.01 ^d^	1.14 ± 0.01 ^e^	51.12
Oleic acid (C18:1)	42.53 ± 0.02 ^a^	3.64 ± 0.02 ^e^	16.82 ± 0.02 ^c^	37.85 ± 0.03 ^b^	13.58 ± 0.03 ^d^	35.88
Linoleic acid (C18:2)	43.73 ± 0.01 ^b^	13.53 ± 0.02 ^e^	48.45 ± 0.03 ^a^	34.65 ± 0.02 ^c^	14.63 ± 0.02 ^d^	42.22
Linolenic acid (C18:3)	0.22 ± 0.00 ^c^	0.01 ± 0.00 ^e^	5.17 ± 0.02 ^a^	0.89 ± 0.01 ^b^	0.09 ± 0.01 ^d^	10.11
Arachidic acid (C20:0)	0.44 ± 0.01 ^a^	0.01 ± 0.00 ^b^	0.01 ± 0.00 ^b^	0.01 ± 0.00 ^b^	-	-
Behenic acid (C22:0)	0.03 ± 0.01 ^a^	0.01 ± 0.00 ^b^	0.01 ± 0.00 ^b^	0.01 ± 0.00 ^b^	-	-
Lignoceric acid (C24:0)	0.01 ± 0.00 ^a^	0.01 ± 0.00 ^a^	0.01 ± 0.00 ^a^	0.01 ± 0.00 ^a^	-	-

^a–e^: Different lowercase letters within the same row indicate statistically significant differences among samples, as determined by Tukey’s multiple comparison test (*p* < 0.05). Means sharing the same letter are not significantly different. BI: Bioaccessibility index.

**Table 4:** Amino acid composition (mg/100 g) of tahini, coconut milk powder, and pea protein powder as well as undigested and IN phase of plant-based protein-enriched tahini powder.

Amino Acid Composition (mg/100 g)	Tahini (Undigested Sample)	Coconut Milk Powder (Undigested Sample)	Pea Protein Powder (Undigested Sample)	Spray-Dried Powder (Undigested Sample)	Spray-Dried Powder (IN Phase)
L-Aspartic acid	1478.00 ± 38.18 ^c^	16.91 ± 0.26 ^e^	8984.00 ± 38.18 ^a^	5333.50 ± 14.85 ^b^	827.50 ± 10.61 ^d^
L-Glutamic acid	4497.00 ± 41.72 ^c^	38.20 ± 0.35 ^e^	12,714.50 ± 41.72 ^a^	7973.00 ± 55.15 ^b^	1760.00 ± 21.21 ^d^
L-Serine	8.44 ± 8.32 ^d^	238.35 ± 8.15 ^c^	N.D.	3809.50 ± 6.36 ^a^	545.00 ± 49.50 ^b^
Glycine	794.50 ± 65.05 ^c^	37.01 ± 1.03 ^e^	3331.00 ± 65.05 ^a^	2142.00 ± 15.56 ^b^	620.00 ± 28.28 ^d^
L-Histidine	1061.50 ± 72.83 ^c^	29.86 ± 0.71 ^e^	2920.50 ± 72.83 ^a^	2265.50 ± 14.85 ^b^	265.00 ± 14.14 ^d^
L-Arginine	480.50 ± 8.49 ^c^	21.85 ± 0.29 ^e^	1695.00 ± 8.49 ^b^	2377.00 ± 11.31 ^a^	342.50 ± 17.68 ^d^
L-Threonine	2062.50 ± 48.08 ^c^	2.42 ± 0.16 ^e^	6191.00 ± 48.08 ^a^	2840.50 ± 23.33 ^b^	1162.00 ± 60.10 ^d^
L-Alanine	617.00 ± 19.80 ^b^	7.49 ± 0.42 ^e^	2264.00 ± 19.80 ^a^	470.00 ± 11.31 ^d^	557.50 ± 45.96 ^c^
L-Proline	1071.00 ± 25.46 ^c^	13.40 ± 0.09 ^e^	3443.00 ± 25.46 ^a^	2466.50 ± 31.82 ^b^	595.00 ± 7.07 ^d^
L-Tyrosine	806.00 ± 7.78 ^d^	43.10 ± 0.34 ^e^	3549.50 ± 7.78 ^a^	1710.00 ± 26.87 ^b^	827.50 ± 38.89 ^c^
L-Valine	727.00 ± 38.89 ^c^	30.02 ± 0.19 ^e^	3063.50 ± 38.89 ^a^	2228.00 ± 39.60 ^b^	702.50 ± 38.89 ^d^
L-Methionine	915.50 ± 21.92 ^b^	26.98 ± 0.31 ^e^	3424.50 ± 21.92 ^a^	241.50 ± 10.61 ^c^	115.00 ± 7.07 ^d^
L-Isoleucine	320.50 ± 4.24 ^d^	1.57 ± 0.17 ^e^	569.00 ± 4.24 ^c^	1847.00 ± 48.08 ^a^	650.00 ± 21.21 ^b^
L-Leucine	791.00 ± 40.31 ^d^	119.95 ± 1.53 ^e^	3768.50 ± 40.31 ^b^	4326.00 ± 38.18 ^a^	1175.00 ± 42.43 ^c^
L-Phenylalanine	1349.50 ± 41.72 ^c^	29.62 ± 0.70 ^e^	5824.50 ± 41.72 ^a^	2929.00 ± 41.01 ^b^	777.50 ± 31.82 ^d^
L-Lysine	829.00 ± 8.49 ^d^	14.91 ± 0.37 ^e^	3738.00 ± 8.49 ^b^	4041.00 ± 42.43 ^a^	1877.50 ± 31.82 ^c^
L-Tryptophan	1236.50 ± 24.75 ^b^	130.54 ± 2.44 ^d^	14,488.50 ± 24.75 ^a^	373.50 ± 2.12 ^c^	79.70 ± 0.35 ^e^

^a–e^: Different lowercase letters within the same row indicate statistically significant differences among samples, as determined by Tukey’s multiple comparison test (*p* < 0.05). Means sharing the same letter are not significantly different. N.D.: Not detected.

**Table 5:** Changes in TPC and antioxidant activities of raw materials and spray-dried powder during INFOGEST digestion and corresponding bioaccessibility indexes.

Samples	The Type of Phases	TPC (mg GAE/100 g)	ABTS Activity (mg TE/g)	FRAP Activity (μmol Fe^2+^/g)	CUPRAC Activity (μmol TE/g Extract)
Tahini	Undigested sample	24.77 ± 0.15 ^c^	32.95 ± 0.36 ^ab^	35.46 ± 0.29 ^b^	41.30 ± 0.15 ^a^
Pea protein powder	Undigested sample	8.79 ± 0.03 ^f^	4.70 ± 0.05 ^f^	6.31 ± 0.13 ^g^	8.42 ± 0.15 ^g^
Coconut milk powder	Undigested sample	18.28 ± 0.13 ^e^	11.35 ± 0.12 ^e^	25.67 ± 0.07 ^d^	18.33 ± 0.12 ^d^
Spray dried powder	Undigested sample	53.47 ± 0.15 ^a^	40.67 ± 0.18 ^a^	53.80 ± 0.18 ^a^	33.22 ± 0.23 ^b^
	Mouth phase	1.86 ± 0.03 ^h^	3.56 ± 0.08 ^g^	3.19 ± 0.18 ^h^	4.38 ± 0.13 ^h^
	Gastric phase	6.36 ± 0.06 ^g^	12.39 ± 0.13 ^d^	9.55 ± 0.12 ^f^	9.36 ± 0.12 ^f^
	IN phase	22.40 ± 0.13 ^d^	14.55 ± 0.18 ^c^	18.46 ± 0.09 ^e^	15.75 ± 0.09 ^e^
	OUT phase	28.14 ± 0.18 ^b^	32.53 ± 0.10 ^b^	28.52 ± 0.09 ^c^	22.67 ± 0.31 ^c^
	BI (%)	44.32	30.90	39.29	40.99

^a–h^: Different lowercase letters within the same column indicate statistically significant differences among samples, as determined by Tukey’s multiple comparison test (*p* < 0.05). Means sharing the same letter are not significantly different. BI: Bioaccessibility index.

**Table 6:** Phenolic compounds and their concentrations in the samples (µg/g).

Identified Phenolics	Tahini (Undigested Sample)	Pea Protein Powder (Undigested Sample)	Coconut Milk Powder (Undigested Sample)	Spray Dried Powder (Undigested Sample)	Spray Dried Powder (IN Phase)	BI (%)
Gallic acid	119.34 ± 0.09 ^a^	32.43 ± 0.02 ^c^	24.63 ± 0.06 ^e^	68.35 ± 2.11 ^b^	26.73 ± 0.03 ^d^	39.11
p-Coumaric acid	0.05 ± 0.01 ^e^	22.75 ± 0.04 ^a^	11.54 ± 0.05 ^b^	3.53 ± 0.07 ^c^	0.72 ± 0.01 ^d^	20.40
Caffeic acid	0.11 ± 0.01 ^d^	1.24 ± 0.01 ^b^	6.85 ± 0.03 ^a^	0.48 ± 0.01 ^c^	0.08 ± 0.01 ^e^	16.67
Syringic acid	0.17 ± 0.01 ^e^	103.52 ± 0.09 ^a^	0.74 ± 0.01 ^d^	17.95 ± 0.05 ^b^	7.24 ± 0.02 ^c^	40.33
Vanillic acid	-	27.85 ± 0.03 ^a^	8.35 ± 0.02 ^b^	5.26 ± 0.06 ^c^	0.83 ± 0.01 ^d^	15.78
Benzoic acid	-	879.68 ± 0.12 ^a^	64.38 ± 0.06 ^c^	137.84 ± 0.08 ^b^	39.82 ± 0.05 ^d^	28.89
Ferulic acid	0.12 ± 0.01 ^e^	53.64 ± 0.08 ^b^	70.34 ± 0.07 ^a^	11.48 ± 0.03 ^c^	1.96 ± 0.01 ^d^	17.07
o-Coumaric acid	0.04 ± 0.01 ^d^	15.87 ± 0.06 ^a^	-	2.93 ± 0.01 ^b^	0.71 ± 0.01 ^c^	24.23
(+)-Catechin	-	46.84 ± 0.07 ^a^	18.49 ± 0.04 ^b^	7.39 ± 0.03 ^c^	3.96 ± 0.02 ^d^	53.59
Quercetin	1.35 ± 0.01 ^e^	113.64 ± 0.04 ^a^	2.61 ± 0.01 ^d^	18.03 ± 0.08 ^b^	8.92 ± 0.02 ^c^	49.47
Apigenin	-	8.47 ± 0.01 ^a^	-	1.35 ± 0.01 ^b^	0.49 ± 0.01 ^c^	36.30
Rutin	0.42 ± 0.01 ^d^	5.34 ± 0.01 ^b^	8.35 ± 0.03 ^a^	1.05 ± 0.01 ^c^	0.31 ± 0.01 ^e^	29.52

^a–e^: Different lowercase letters within the same row indicate statistically significant differences among samples, as determined by Tukey’s multiple comparison test (*p* < 0.05). Means sharing the same letter are not significantly different.

## Data Availability

The original contributions presented in this study are included in the article. Further inquiries can be directed to the corresponding author.
